# Depression and Anxiety in Patients With a History of Traumatic Brain Injury: A Case-Control Study

**DOI:** 10.7759/cureus.27971

**Published:** 2022-08-13

**Authors:** Dania A Al-Kader, Chimaoge I Onyechi, Ijeoma V Ikedum, Abdul Fattah, Shumaila Zafar, Sadaf Bhat, Mohammad A Malik, Nimarta Bheesham, Laila Tul Qadar, Mustafa Sajjad Cheema

**Affiliations:** 1 Radiology, Liaquatian Academic and Research Society, Hyderabad, PAK; 2 Psychiatry, Liaquatian Academic and Research Society, Hyderabad, PAK; 3 Internal Medicine, Liaquatian Academic and Research Society, Hyderabad, PAK; 4 Internal Medicine, Indus Medical College, Tando Muhammad Khan, PAK; 5 Internal Medicine, Mayo Hospital Lahore, Lahore, PAK; 6 Internal Medicine, Liaquat University of Medical and Health Sciences, Jamshoro, PAK; 7 Internal Medicine, Dow University of Health Sciences, Karachi, PAK; 8 Internal Medicine, Combined Military Hospitals (CMH) Lahore Medical College, Lahore, PAK

**Keywords:** 9-item patient health questionnaire (phq-9), gad-7, depression, clinical anxiety, anxiety

## Abstract

Background

The burden of psychiatric illness following a head injury may have implications on the disease prognosis. The present study evaluated the association of depression and anxiety with traumatic brain injury (TBI).

Methods

A case-control study was conducted in Karachi, Pakistan, from 2nd July 2021 and 30th January 2022, including individuals of age 18 and above of both genders, with or without a mild head trauma history, forming the case and control groups, respectively. Individuals with previous head trauma/congenital neurological dysfunction were excluded. A mental health assessment of the participants was carried out with two scales, the Generalized Anxiety Disorder-7 (GAD-7) scale and the Public Health Questionnaire-9 (PHQ-9) scale. Other parameters like age, gender, socioeconomic status, education status, and comorbidities were also documented.

Results

A total of 62 participants were enrolled with 31 cases and 31 controls. The majority were males aged between 18 and 39 years. About 29% of the patients with a history of mild TBI had moderate to severe depression while only 22.6% of them did not have depression or had minimal depression. We found that about 29.3% of patients with TBI had severe anxiety as compared to the only two healthy controls. The majority of the control group participants did not have anxiety.

Conclusion

Traumatic head injuries and their long-term side effects can predispose patients to a myriad of psychiatric comorbidities. In this study, we found definitive evidence that both anxiety and depression had a significantly higher incidence in cohorts that suffered from mild TBI. However, we recommend large-scale and multicenter studies in the future to explore these relationships more thoroughly and comprehensively.

## Introduction

Traumatic brain injury (TBI) may result in a range of symptoms that affect an individual’s cognition and psychological well-being [1]. Most cases of TBI globally and within the United States result in symptoms that are mild in severity, i.e. mild TBI (MTBI). These are commonly known as concussions. The symptoms that develop after TBI are usually temporary [2]. The range of neurologic symptoms experienced consists of psychiatric symptoms (e.g. mood disorders such as depression, anxiety, and irritability), sensory and somatic complaints (e.g. sleep-related issues, headaches, blurring of vision, and dizziness), and cognitive and behavioral issues (e.g. patient-perceived memory issues, difficulty handling information, and attention and concentration issues) [1-5].

The effects of TBI on cognition may be considered to be the most damaging [6]. These cognitive symptoms lead to greater enduring morbidity than the physical symptoms of TBI. Persistent deficiencies are elicited in areas of cognition such as concentration, anamnesis, and executive functioning [2-6].

Belanger et al. in their meta-analysis studied the long-term effects of concussions (MTBI) on cognition [7]. They studied various, defined areas of cognition. Visuospatial functioning, concentration, and language suffered a moderate decline whilst speech and memory were greatly affected within 90 days of the TBI. Thereafter, the degree of cognitive decline and functioning depended upon the patient’s sample group, i.e. whether they were in a healthcare environment or prospectively studied, and if they had been actively engaged in legal proceedings [7].

Aside from cognitive and neurologic deficits, individuals suffering a TBI of any severity are more likely to develop long-term psychiatric disabilities such as mood disorders, i.e. depression and anxiety, and post-traumatic stress disorder (PTSD) [8], due to the psychological trauma of the incident. Mental distress following TBI has been documented in the literature to have been inconsistent [9]. It is important to note that the factors surrounding the TBI incurred as well as those after injury affect the presentation of the ensuing psychological distress.

Despite having a high incidence rate of TBIs owing to a huge road traffic injury burden [10-11], the literature from local regions is quite scarce. Thus, considering the dearth of literature, the current study was undertaken with the main goal to evaluate the severity of depression and anxiety in patients with traumatic brain injury.

## Materials and methods

A case-controlled study was undertaken at the Department of Neurosurgery, Liaquat University of Medical & Health Sciences (LUMHS), between 2nd July 2021 and 30th January 2022 after obtaining approval from the Institutional Review Board of LUMHS (LUMHS/REC/1025).

All patients who had presented with mild traumatic brain injury between the age of 18 and 65 years were eligible to be included. The healthy controls were chosen from the community and matched to the patient's sociodemographic characteristics. The controls could be the attendant (friend or relative) of the patient to ensure similar socio-demographics. The exclusion criteria comprised individuals with intellectual/mental disabilities from birth, neurodegenerative diseases, and individuals having a disability rendering them sedentary and bedbound prior to the injury.

The technique used for the recruitment of participants was non-probability convenience sampling. Using the OpenEPI electronic calculator ( www.OpenEpi.com) for comparing two means, the sample size was determined. The mean value for the depression test scores for head injured patients and controls was 36.25 ± 8.4 and 31.14 ± 5.54, respectively, as reported by Schoenhuber and Gentilini [12]. Using a confidence level of 95%, and a power of 80%, a total sample size of 62 was obtained, with 31 in each group.

A mental health assessment of the participants was carried out with two scales, the Generalized Anxiety Disorder-7 (GAD-7) scale and the Public Health Questionnaire-9 (PHQ-9) scale. Participants in both case and control groups were assessed. GAD-7 is accurate and dependable in determining the intensity of generalized anxiety disorder, and PHQ-9 is a relatively newer scale used in primary care for the assessment of mental illnesses including depression. Other parameters like age, gender, socioeconomic status, education status, and comorbidities were also documented.

SPSS version 25.0 (IBM Corp., Armonk, NY) was used to evaluate the data. For mean and standard deviation, descriptive statistics were computed. Categorical variables were denoted as frequency and proportions. For correlation between psychological disabilities and other sociodemographic variables, including gender, a chi-square test was utilized. A p-value of less than or equal to 0.05 was considered statistically significant.

## Results

A total of 62 participants were enrolled with 31 cases and 31 controls. The majority were males aged between 18 and 39 years. Table 1 illustrates the specific proportions of different sociodemographic parameters in both groups. There was no statistically significant difference in these demographic parameters between the case and control groups.

**Table 1:** Sociodemographic characteristics of case and control groups

Characteristics	Case	Control	Total N (%)	P-value
Age groups				
＜40 years	20 (64.5%)	18 (58.1%)	38 (61.3%)	0.602
≥ 40 years	11 (35.5%)	13 (41.9%)	24 (38.7%)	
Gender				
Female	11 (35.5%)	6 (19.4%)	17 (27.4%)	0.155
Male	20 (64.5%)	25 (80.6%)	45 (72.6%)	
Residence				
Urban	29 (93.5%)	30 (96.8%)	59 (95.2%)	0.554
Rural	2 (6.45%)	1 (3.23%)	3 (4.84%)	
Education Status				
Secondary High School	16 (51.61%)	14 (45.16%)	30 (48.39%)	0.875
Bachelors	14 (45.16%)	16 (51.61%)	30 (48.39%)	
Postgraduate	1 (3.23%)	1 (3.23%)	2 (3.23%)	
Ethnicity				
Sindhi	3 (9.68%)	3 (9.68%)	6 (9.68%)	0.639
Punjabi	6 (19.35%)	4 (12.9%)	10 (16.13%)	
Pushtoon	2 (6.45%)	1 (3.23%)	3 (4.84%)	
Urdu Speaking	19 (61.29%)	19 (61.29%)	38 (61.29%)	
Other	1 (3.23%)	4 (12.9%)	5 (8.06%)	

We found that patients who had a history of mild traumatic brain injury (MTBI) more frequently suffered from depression and anxiety (Table 2 and Table 3). We found that about 29.3% of patients with MTBI had severe anxiety as compared to the only two healthy controls (Table 2). The majority of the control group participants did not have anxiety.

**Table 2:** Association of anxiety with mild traumatic brain injury

Anxiety	Control	Case	p-value
Normal	15 (48.4%)	8 (19.3%)	0.03617
Mild Anxiety	8 (25.8%)	10 (32.3%)	
Moderate Anxiety	6 (19.4%)	6 (19.4%)	
Severe Anxiety	2 (6.4%)	7 (29.3%)	

**Table 3:** Association of depression with mild traumatic brain injury

Depression	Control	Case	p-value
No Depression or Minimal Depression	17 (54.8%)	7 (22.6%)	0.049
Mild Depression	7 (22.6%)	9 (29.0%)	
Moderate Depression	4 (12.9%)	6 (19.4%)	
Moderate to Severe Depression	3 (9.6%)	9 (29.0%)	

Table 3 illustrates the association between depression and MTBI. It was found to be statistically significant (p = 0.04). About 29% of patients with a history of MTBI had moderate to severe depression while only 22.6% of them did not have depression or had minimal depression.

## Discussion

A rapid, abrupt, and excessive mechanical injury to the head may result in a sudden decline in brain functionality; this is a traumatic brain injury (TBI). These injuries are quite common and usually demand emergency medical/surgical management [13]. TBIs are often acknowledged as “head injury”, “concussion”, “intracranial bleed”, and “contusion”. In low-income and middle-income nations, TBI greatly impacts the economy. It is reported that 75% are secondary to falls and 50% are due to road accidents [14].

Rosenbaum PE et al. recognized TBI as a complex condition. According to their comprehensive research, individuals suffering from moderate-to-severe TBI carry an elevated risk of disordered behavior and cognition. These impairments hinder patients’ physical and occupational rehabilitation and social re-integration, resulting in an overall poorer quality of life (QoL) [15]. The findings from Schoenhuber and Gentilini also highlighted the relationship between mild head trauma and the risk of later developing certain psychiatric ailments [12]. The findings of our study correspond with the current body of literature.

Cognitive disability and psychological distress were both observed in a cohort of 74 patients by Payne et al., with participants documenting reduced feelings of fulfillment with life and reduced well-being [16]. Bivariate associations between almost all the variables of well-being were significant. Correlations were as anticipated. Two more novel scales of well-being were utilized in this study, and these correspond well with the more recognized ones. Participants who were out of work but wished to remain productive weren’t satisfied with their QoL and their well-being was decreased. These findings demonstrated the importance of improving satisfaction and productiveness for this population with targeted programs. This study further stresses the need for better comprehension of the mental health outcomes following TBI [16].

The findings of Lavoie et al. illustrate that the greater prevalence of mild depression in women in the general populace is not reflected in patients suffering moderate-to-severe TBI [17]. Thus, prompt recognition of psychological symptoms, diligent screening, and apt management for all are essential in the betterment of mental health and QoL and in advancing recovery [17]. 

A study by Barker-Collo et al. evaluated the psychological symptoms of depression and anxiety in participants suffering from mild TBI (Glasgow Coma Scale (GCS) 13-15), all aged ≥ 15 years at the time of trauma, over four years [18]. In this set of patients, both depression and anxiety seemed to significantly reduce as time passed. This was reflected by reducing the mean anxiety score and total score for depression. The prevalence of anxiety was 3.7% to 29.5% and that of depression was 0% to 7.7% while that of both together was 10.2% to 20.7%. At all instances of evaluation, most participants (28% to 49%) reported neither. Their results speculated that disease progression takes numerous directions as time progresses. It was concluded that adequate screening for mental health symptoms, i.e. anxiety and depression, is necessary after mild TBI [18]. Additionally, it is inappropriate to label patients that initially do not report these symptoms as unlikely to develop depression and anxiety later during their recovery.

Since the year 2019, the world has been facing the disastrous Coronavirus disease 2019 (COVID-19) pandemic, which is associated with psychological scarring and increased risk of depression among patients [19]. Those with depression and sleep disorders expressed greater COVID-19-related trauma. The impact of co-morbid chronic physical diseases on COVID-19 trauma, particularly depression, perceived susceptibility to COVID-19 and reported that the probability of recovery in the event of COVID-19 infection was mediated by anticipated physical and mental wellbeing [19].

Despite several strengths, the study had some limitations. Due to the limited sample size, the study findings could not be generalized. Furthermore, another limitation was the small duration of follow-up due to resource constraints. Therefore, we recommend that further large-scale studies in multiple centers should be conducted.

## Conclusions

It was found that a significantly higher proportion of people who had a mild traumatic brain injury (MTBI) later developed depression or anxiety. Thus, it is strongly recommended that patients with a history of MTBI should be screened for psychiatric comorbidity, especially depression and anxiety. Furthermore, large-scale, multicenter, longitudinal studies are recommended to explore the relationship between neuropsychiatric comorbidity with TBI in patients.
